# Real-Time Access to Electronic Health Record via a Patient Portal in a Tertiary Hospital: Is it Harmful? A Retrospective Mixed Methods Observational Study

**DOI:** 10.2196/13622

**Published:** 2020-02-11

**Authors:** Savannah IR van Kuppenveld, Harmieke van Os-Medendorp, Nicole AM Tiemessen, Johannes JM van Delden

**Affiliations:** 1 University Medical Center Utrecht Utrecht Netherlands; 2 University Medical Center Utrecht, Department of Dermatology and Allergology Utrecht Netherlands; 3 Saxion University of Applied Sciences, School of Health Deventer/Enschede Netherlands; 4 University Medical Center Utrecht, Department Information Technology Utrecht Netherlands; 5 University Medical Center Utrecht, Department of Medical Humanities, Julius Center Utrecht Netherlands

**Keywords:** patient portals, communication, health services research, information technology

## Abstract

**Background:**

The rapid implementation of patient portals, through which patients can view their electronic health record, creates possibilities for information exchange and communication between patients and health care professionals. However, real-time disclosure of test results and clinical reports poses a source of concern.

**Objective:**

This study aimed to examine negative experiences resulting from real-time disclosure of medical information through a patient portal.

**Methods:**

Data were collected over a 2-year period in 4 datasets consisting of incidents reported by health care professionals, complaints of patients, patient issues at a portal helpdesk, and a survey among health care professionals. Incidents, complaints, issues, and answers on the survey were counted and analyzed through an iterative process of coding.

**Results:**

Within the chosen time frame of 2 years, on average, 7978 patients per month logged into the portal at least once. The amount of negative incidents and complaints was limited. A total of 6 incidents, 4 complaints, and 2506 issues at the helpdesk concerning the patient portal were reported, of which only 2, 1, and 3 cases of these respective databases concerned real-time disclosure of medical information through the patient portal. Moreover, 32 out of 216 health care professionals reported patients that had negative experiences with real-time disclosure. Most negative consequences concerned confused and anxious patients when confronted with unexpected or incomprehensible results.

**Conclusions:**

Real-time access through a patient portal did not substantially result in negative consequences. The negative consequences that did occur can be mitigated by adequate preparation and instruction of patients concerning the various functionalities of the patient portal, real-time disclosure of test results in particular, and can also be managed through educating health care professionals about the patient portal and making adjustments in the daily practice of health care professionals.

## Introduction

### Electronic Health and Patient Portals

Electronic health (eHealth) is defined by the World Health Organization (WHO) as “the use of information and communication technologies (ICT) for health” [1]. eHealth and mobile health are encouraged by the WHO to strengthen health care organizations to increase access to care and health information and to improve safety and quality of care [2]. Access to personal health information in a medical file can be offered via a patient portal. In the Netherlands, it is the ambition of the Dutch Ministry of Health, Welfare and Sport that most patients have access to their medical data, can share personal data, and can use these data to improve their personal lifestyle. In addition, insight into medical data will contribute to transparency in health care, better informed patients, and shared decision making [3]. In the Netherlands alone, the number of hospitals that provide access to a patient portal has doubled in 2 years’ time from July 2016 to July 2018 [4], and upcoming legislation concerning Web-based access to one’s medical data will likely increase even further. These ambitions are also seen in the United States, eg, OpenNotes [5] and My HealtheVet, a Web-based patient portal of the Veteran Health Administrations [6], and in European countries such as Sweden, the United Kingdom, and Germany [7-9].

The medical dictionary [10] defined a patient portal as ‘a domain in an electronic health record (EHR) that allows patients to access their records or communicate with their healthcare providers.’ Patient portals are distinguished from personal EHRs in terms of ownership: a patient portal is mostly tethered to a health care organization, whereas a personal health record is untethered but owned by the patient and may include information that is not part of a medical record [11,12]. The patient portal provides patients insight into (parts of) their EHR and test results and can also offer a wide variety of other functionalities such as communication with professionals, the possibility to make appointments, and request prescription refills and can also provide patient education [12]. Owing to the absence of guidelines, the ways in which these functionalities are effectuated are diverse. One of these functionalities is the disclosure of test results. The time taken for medical information that enters the EHR to be accessible to the patient through the portal varies significantly. There are, eg, portals where results are released manually, portals that have a built-in delay of 48 hours, portals where timing of release is adjusted to particular results, and portals that release all results in real time [13,14].

### Impact of a Patient Portal

Online access to a patient portal has shown to positively impact patient engagement by making patients active participants in their care, and it also supports patient empowerment by enabling patients to be better informed and making them feel more in control [13,15-18]. Furthermore, access to a patient portal can also improve the patient-doctor relationship [14,15,19,20]. Although there is increasing evidence of the positive impact of patient portals, concerns of both physicians and patients about possible negative consequences of releasing test results before consultation to a health care professional remain. Real-time disclosure enables patients to look into their data irrespective of whether health care professionals have had a chance to look into it as well. This eliminates physicians as the sole intermediaries of medical information, including possibly alarming information. Studies show that physicians are uncomfortable with direct release of test results, uttering that it can cause patient anxiety [13,21] and confusion [21]. Although physicians seem to worry more about these potential consequences [22], patients themselves are not exempt from them either. Some patients are concerned about the inability to interpret the nature and relevance of their medical data, which may cause anxiety and confusion [22,23].

### Aim

Although these concerns are reflected in various studies, little is known about the actual impact of access to a patient portal on patients, let alone real-time disclosure. We, therefore, conducted a study aiming to examine negative experiences resulting from real-time disclosure of medical information and test results via a patient portal.

## Methods

### Study Design

This retrospective mixed methods observational study used 4 preexisting databases to examine the negative experiences of health care professionals and patients at University Medical Center Utrecht (UMCU), a tertiary hospital for adults and children. The databases covered a 2-year period, starting on September 1, 2015, 6 months after implementation of the patient portal for adults and 1 week after implementation of the patient portal for (authorized representatives of) children, and ending on September 1, 2017. As the implementation of the patient portal was carried out in 2 phases, we chose to start our analysis of the data upward of September 1, 2015, to maintain a clear time line of our data. The Medical Research Involving Human Subjects Act (in Dutch: Wet-Medisch-wetenschappelijk Onderzoek met mensen) did not apply to this study, and therefore, an official approval of this study was not required, which was confirmed by the Medical Research Ethics Committee Utrecht (protocol number 17.759/C).

### System Description

The patient portal “My UMC Utrecht” is available to all patients of UMCU. The patient portal was implemented in February 2015 for adults and in August 2015 for (authorized representatives of) children. The patient portal can be accessed by computer, mobile phone, or tablet (iPad). The hospital provided several means to inform patients about the patient portal. There was an instruction on the hospital website, posters and banners were placed in the hospital building, flyers were disseminated by administrative assistants, and health care professionals and patients were sent a flyer after their first appointment. In addition, some health care professionals provided information to their patients on consultation. The patient portal allows patients to access parts of their EHR. The information shown in “My UMC Utrecht” is disclosed in real time. There is no delay between the information in the EHR and the patient portal, and no alterations have been made concerning phrasing, ie, information that enters the EHR is directly visible in the patient portal regardless of whether health care professionals have viewed the information. Before entering the result section of the portal, a pop-up is shown reminding patients of the real-time disclosure (see Multimedia Appendix 1).

Access to a patient portal entails access to clinical notes and scheduled and previous appointments. It also provides the possibility to request repeat prescriptions, fill in questionnaires for both care and research purposes, make personal notes, and communicate with health care professionals through electronic consults (e-consults). Patients can send an e-consult by selecting the department of the physician they want to communicate with. The administrative assistant of that specific department will forward the e-consult to the right physician. E-consults are to be answered within 2 to 3 working days, either by an administrative assistant communicating that the message has been forwarded to the patient’s physician or by the physician himself/herself.

“My UMC Utrecht” includes test results such as laboratory results and reports, radiology reports, pathology reports, and daily reports. Reports registered by interns are visible only after they have been validated by a supervisor. Health care professionals have the possibility to manually close the patient portal stating the reason. Professionals of the intensive care unit temporarily close the patient portal for their patients. Furthermore, because of incompatibility of software with the patient portal, clinical notes from the department Woman and Baby, emergency room reports, ophthalmological diagnostics, and medical images are not available via the patient portal. Concerning the latter, patients do have access to the radiologist’s written interpretation. Finally, there is a field in the EHR where physicians can document personal notes that are not shown in the patient portal.

### Data Collection

The databases that were used for analysis were as follows: patient care incident reports reported by health care professionals, complaints of patients at the complaint commission, surveys among health care professionals and administrative assistants, and summaries of issues concerning the patient portal reported by helpdesk employees. These 4 anonymized databases were chosen for analysis because of their nature to capture adverse consequences of the patient portal.

The patient care incident reports were registered by health care professionals according to a fixed format of 4 questions. These reports were received from the secretary of the commission that registers notifications of incidents in patient care (NIP), ie, the NIP commission. The complaints of patients concerning the patient portal were reported at the hospital complaint commission. These complaints were received from the complaint commission. The negative experiences with real-time disclosure of the patient portal were deducted from a digital survey that was disseminated via email among professionals and administrative assistants in May and June 2017 by the managers of the hospital’s 12 departments. It is unclear how many health care professionals and administrative assistants were reached by this survey. The surveys were received from 1 of the authors of this study, who had set up the questions together with members of the former patient portal board in the context of a previous hospital assessment concerning the patient portal. The summaries of issues concerning the patient portal were registered at a helpdesk for patients. Patients could contact the helpdesk via phone, email, or by visiting the helpdesk counter. Issues include questions, complaints, remarks, and requests for help of patients, and occasionally employees, registered between October 31, 2015, and September 12, 2017, by 3 helpdesk assistants. These summaries were received from the product manager of the patient portal.

### Data Analysis

The data were analyzed both qualitatively and quantitatively. The databases were analyzed qualitatively by identifying and coding themes and quantitatively by counting how many times a certain theme or problem was addressed. The databases were imported in the software program NVivo Pro 11 (QSR International) to facilitate counting and coding.

The 4 databases were analyzed in a slightly different way. In the NIP database, incidents that were falsely labeled “patient portal” were filtered out, eg, incidents that concerned the EHR itself. A total of 57 incidents were excluded because they concerned incidents that were not related to the patient portal. Themes were identified out of the remaining incidents by examining the main topic of the individual incidents. Subsequently, the amount of incidents within each theme was counted. Similarly, the complaints were thematically analyzed and counted. The surveys were analyzed by counting the amount of respondents that reported having had negative experiences with the patient portal. When respondents did not answer “yes” to the question of having had negative experience(s) but did describe 1 or more negative experience(s), they were coded as if the answer to the first question was “yes.” The reported negative experiences were analyzed thematically by coding the topic(s) the respondents addressed. Hereafter, it was counted how many times the identified themes were addressed by the respondents. Similarly, the helpdesk issues were coded by the topic(s) addressed and subsequently counted per theme.

The quality of the coding schemes was ensured by the iterative process of going back and forth within the databases to ascertain the appropriateness of the ascribed themes. Furthermore, the individual items within each theme were examined to determine whether they truly belonged within that theme. Hereafter, the items that were coded as “disclosure of information” were chosen for further analysis. Through axial coding, subthemes were identified and, if evident from the item, also the patients’ emotion. The reports were coded by 1 person (SK), with regular outcome discussions within the research group.

## Results

### Patient Visits

In 2015, the hospital received 99,326 outpatient visits of new patients and hospitalized 29,676 patients; in 2016, these numbers were 94,696 and 31,342, respectively; and in 2017, the numbers were 93,983 and 30,171, respectively. Within the chosen time frame of 2 years, 190,000 patients had access to the portal, and, on average, 7978 patients per month logged into the portal at least once. In addition, there seems to be an increase in the number of patients that logged into the portal at least once (see Multimedia Appendix 2).

### Notifications of Incidents in Patient Care

In these 2 years, 63 incidents were reported by employees of the UMCU, which were categorized as “patient portal” by the health care professional that registered the incident. After looking closely at these incidents, only 6 incidents truly concerned (the use of) the patient portal. As shown in Table 1, 2 incidents have been reported concerning the real-time disclosure of information through the portal, 2 incidents concerned faulty information shown in the patient portal, 1 incident concerned privacy and security of patients and their data, and 1 incident concerned e-consults.

The 2 incidents that concerned real-time disclosure of information described patients acquiring information through the patient portal before consulting a health care professional. One incident concerned a patient who was unaware of the real-time aspect of disclosure of results and accidentally saw the results of a magnetic resonance imaging scan of his brain. The patient was startled by the possibility of seeing potential adverse outcomes. The other incident concerned parents who noticed an appointment that had not been announced and of which the nature was unclear. This caused the parents to worry about whether this indicated their child was scheduled for surgery or not.

Of the 2 incidents that described patients discovering faulty information in their medical record, one concerned a patient that noticed 1 of the reports contained a medical history that was not hers (also reported by a respondent in the survey). The other incident concerned a patient who noticed 2 medical letters were sent to the wrong address.

The incident about privacy and security concerned parents who received access to the medical record of someone else’s child.

The incident about e-consults concerned an inadequate follow-up of a potential urgent e-consult.

**Table 1:** Themes of incidents, complaints, and helpdesk issues concerning the patient portal.

Themes	Number of notifications of incidents in patient care (n=63)	Number of complaints addressed at complaint commission (n=4)	Helpdesk requests^a^ (n=2673), n (%)
Patient portal issues	6	4	2506 (93.75)
Real-time disclosure	2	1^b^	3^b^ (0.00)
Discovery of faulty information by a patient	2^c^	—^d^	21 (0.78)
Results/reports not in the patient portal	—	1	133 (4.97)
Security and privacy	1	—	18 (0.67)
(Follow-up) electronic consult	1	—	55 (2.06)
Logging on	—	1	184 (6.88)
Difficulty acquiring access to the patient portal	—	1	634 (23.72)
Other (eg, technical issues, navigation, and provision of information)	—	—	1524 (57.01)
Not patient portal related	57	—	167 (6.24)

^a^One respondent can address multiple situations and/or experiences; therefore, the sum of the column adds up to more than its total.

^b^Complaint registered by both complaint commission and helpdesk.

^c^One of these incidents is also reported by a respondent in the survey for health care professionals.

^d^This theme did not occur in the database.

### Complaint Commission

A total of 4 complaints were issued at the complaint commission. Moreover, 1 complaint concerned real-time access to the patient portal and was filed by the daughter of a terminally ill patient. According to the daughter, her father panicked after looking into his lab results, which indicated that his condition had deteriorated. In her opinion, the pop-up preceding the entrance of the result section, which reminded patients of the real-time disclosure, laid too much responsibility on patients and their next of kin. In another complaint, it is issued that medical images are not accessible via the patient portal. Another complaint concerned parents who were unable to acquire access to their child’s patient portal. There was also a complaint made by a patient who could not access the patient portal. Due to his medical condition he was unable to use a mobile phone, which is required for the log-on procedure of the patient portal.

### Survey Health Care Professionals

It is unknown how many health care professionals were reached by the questionnaire; therefore, we are unable to determine the response rate. A total of 288 health care professionals filled in the questionnaire, out of which 216 answered 1 or more of the questions regarding negative experiences of patients with the patient portal. Respondent characteristics are shown in Table 2. As shown in Table 3, 50 respondents (50/216, 23.1%) reported having negative experiences with disclosure of medical information through the portal, and 32 respondents (32/216, 14.8%) reported having had negative experiences with the real-time aspect of disclosure in particular. A total of 16 respondents (16/216, 7.4%) reported negative experiences because of the inability of patients to comprehend or interpret test results. According to the respondents, this resulted in confusion, worry, or anxiety in patients. Moreover, 9 respondents (9/216, 4.2%) reported negative experiences of patients because of the unavailability of health care professionals short after seeing test results causing patients to worry and feel anxious, impatient, or angry. In addition, 9 respondents (9/216, 4.2%) reported worry, dissatisfaction, and panic of patients without further specifying the context in which these emotions arose.

Furthermore, 21 health care professionals (21/216, 9.7%) reported negative experiences with patients who were dissatisfied with the content of clinical notes. This concerned patients who did not agree with the phrasing of their doctor.

A total of 2 respondents (2/216, 0.9%) stated that patients discovered faulty information in their health record. One concerned a patient that saw the report of another patient that had been wrongly registered in her health record (also described in the NIPs). The other respondent described a discrepancy between the appointment communicated in an invitation letter and the appointment shown in the portal.

Finally, 1 respondent (1/216, 0.0%) reported a patient for whom it was not possible to view certain results.

**Table 2:** Characteristics of respondents in the survey among health care professionals.

Respondent characteristics		Values
**Gender, n (%)**		
	Female	159 (73.6)
	Male	52 (25.0)
	Missing	5 (2.3)
Age (years), mean (range)		42.9 (20-64)
Years in practice, mean (range)		10.1 (0-32)
**Position, n (%)**		
	Health care professional	168 (77.7)
	Administrative assistant	48 (22.2)
**Department, n (%)**		
	Internal medicine and dermatology	65 (30.0)
	Surgery	49 (22.7)
	Brain	40 (18.5)
	Children	34 (15.7)
	Woman and baby	8 (3.7)
	Vital functions	7 (3.2)
	Heart and lungs	5 (2.3)
	University Medical Center Cancer Center	2 (0.9)
	Radiology	0 (0.0)
	Biomedical genetics	0 (0.0)
	Julius Center for health sciences	0 (0.0)
	Laboratory and pharmacy	0 (0.0)
	Missing	6 (2.7)

**Table 3:** Themes of negative experiences of patients with the patient portal reported by health care professionals.

Theme		Survey health care professionals (n=216)^a^, n (%)
Negative experience with disclosure		50 (23.1)
**Negative experience with** **real-time** **disclosure**		**32 (14.8)**
	Due to inability to interpret results/absence of explanation	16 (7.4)
	Due to unavailability of health care professionals	9 (4.2)
	Unknown cause	9 (4.2)
Patient dissatisfaction with reports		21 (9.7)
Discovery of faulty information by a patient		2^b^ (0.9)
No access to content		1 (0.0)

^a^One respondent can address multiple situations and/or experiences; therefore, the sum of the column adds up to more than its total.

^b^One of these negative experiences was also registered in a notifications of incidents in patient care.

### Patient Helpdesk

Out of the 2673 requests or issues reported at the helpdesk that were labeled “patient portal,” 2506 (93.75%) truly concerned the patient portal, others concerned issues with EHR or issues unrelated to the patients’ health record. Moreover, 3 issues (3/2673, 0.0%) concerned patients that had a negative experience with disclosure of test results in real time. One of these was also sent to the complaint commission and has been described earlier. Another issue concerned an employee who reported that a patient got extremely upset and got into trouble as a result of seeing test results. The summary does not specify the exact circumstances. Another issue concerned a patient that explicitly requested to not see test results or reports in real time because she thought the inability to interpret the medical jargon would result in speculation.

A total of 21 patients (0.79%) contacted the helpdesk because they discovered faulty information in their portal.

Furthermore, 133 requests (133/2673, 4.97%) concerned patients that commented on the unavailability of results and/or reports in the patient portal. Moreover, 11 requests (11/2673, 0.00%) concerned patients that asked why their results were not disclosed yet and questioned whether disclosure had been delayed. In addition, 122 patients (122/2673, 4.56%) noted that some results or reports were not shown in the portal. These results and reports concerned specific types of information that are not incorporated into the patient portal altogether such as medical images and information that is processed via systems that are incompatible with the patient portal (for specifics, refer to the System Description section).

In addition, 634 (634/2673, 23.72%) patients reported difficulty acquiring access to the patient portal. It was not always clear why some patients experienced this difficulty. Patients that did include what their specific difficulty entailed mentioned difficulty with the verification procedure via SMS and the digital identity verification system, incorrect authorization for the portal, and absence of an ID verification date.

## Discussion

### Principal Findings

Our study shows that both patients and health care professionals report having had negative experiences in relation to the real-time aspect of disclosure of medical information and test results via a patient portal. Reported negative experiences are patient anxiety and confusion; however, the prevalence of these negative experiences is relatively low and manageable.

### Comparison With Literature

The relatively low number of negative experiences resulting from real-time disclosure was also reported in comparative studies. A qualitative study that examined experiences of primary care practitioners and patients who received abnormal test results also found that anxiety resulting from direct access to test results seems to be limited [13]. Another study shows that there is no overall difference in anxiety levels in patients receiving a normal or abnormal result regarding direct-to-patient disclosure of mismatch repair screening for Lynch syndrome [24].

Others show that anxiety is also limited when patients access a patient portal without real-time disclosure. Moreover, 2 studies among cancer patients showed that Web-based access to medical records did not increase anxiety levels or generate substantial anxiety [14,25]. Another study examining the experiences of primary care practitioners and patients with abnormal test result notification through patient portals reported that participants expressed concern but few indicated having had negative experiences with the portal [23]. These studies also showed that patients want access to both normal and abnormal test results [14,23].

We found that negative experiences of patients with real-time disclosure mostly originate from the inability to interpret test results. This is in accordance with findings of a study among patients and physicians that use the MyPreventiveCare portal, which was designed to activate and engage patients in preventive care. They found that patients find it difficult to interpret laboratory data [26]. Moreover, 1 study among kidney transplant patients shows that when result presentation is visually assisted (by coloring, placement, and charts), misinterpretation is still high [27].

Contrary to these studies were the results from studies concerning clinical notes. Furthermore, 1 study among primary care practitioners and their patients [20] and 1 study among adult patients and parents of pediatric patients [19] found that most patients find the clinical notes relatively easy to understand and that access to these notes could help reduce confusion and enhance understanding of test results as well as the reasons behind tests.

Although other studies found that negative experiences can arise from discovery of errors, inconsistencies, or missed test results [14,16], the patient portal can contribute to enhancement of the quality of care by enabling patients to detect errors or inconsistencies and have them corrected, thereby safeguarding their EHR from error. In addition, the portal could also prevent missing test results and secure follow-up. These notions are illustrated by patients in our study who noted that their portals contained faulty information and patients that enquired about results and reports that were not (yet) accessible via the portal. This is supported by other studies that found that patient portals enable patients to discover errors or missed test results in their EHR [13,21,26].

We believe that real-time disclosure of medical information can be in accordance with the provision of good care. Good guidance of the entire process from test request to test result delivery is essential. Health care professionals should anticipate what the patient might see and should be available for questions (by consult) within a reasonable amount of time. Health care professionals can help mitigate anxiety and confusion by adopting strategies such as allocating time during consultation to explain how and when medical information becomes available and what kind of results patients can expect [15]. In addition, the period between release of results and their interpretation should be brought to a minimum. Quick interpretations of health care professionals accompanying the results in the patient portal could help reduce or eliminate patient anxiety [13]. Health care professionals as well as students should be educated about the patient portal and real-time disclosure, in particular, to help them acquire and practice skills for good guidance of their (prospective) patients.

Medical paternalism can stand in the way of the patients’ right to access their medical information where and whenever they want and to be notified timely. Good guidance will enable good care without withholding patients from the possibilities this new era of technology has to offer.

Furthermore, there is reason to believe that hesitation or reluctance to adopt real-time disclosure through patient portals is motivated by status quo bias [28,29]. The preference for current practices in health care can originate from the uncertainty or fear of the risk associated with this new form of communication as well as from an underestimation of the additional value over and above the current state of affairs. The results of this study show that the reality of real-time disclosure does not seem to live up to the fears of presumed severe adverse consequences. In addition, current practice is not as advantageous as we might want to believe. In current practice, patients have to wait several days, if not longer, to receive the results of diagnostic procedures. The uncertainty in awaiting these results can have adverse effects on patients. For example, 1 study showed that waiting for radiology test results negatively affects patients’ state of mind, with anxiety being the most common emotional state [30]. Furthermore, another study showed that women awaiting breast biopsy and diagnosis experienced high levels of anxiety, which was shown to be a greater stressor than awaiting the riskier invasive treatment of known cancer [31].

### Limitations

It is unlikely that severe adverse consequences with the patient portal have not been picked up by any of the databases. The databases register adverse consequences by design and encompass experiences reported by both patients (complaint commission and helpdesk) and health care professionals (NIPs and survey). However, we are aware that we do not capture all negative experiences, as patients can chose to refrain from seeking contact. Furthermore, even if patients did seek contact, the involved health care professional might not have been reached by the survey. Moreover, the amount of patients that contacted the helpdesk with difficulties concerning the log-in procedure indicates that less patients acquired access to their portal than desirable. In addition, patients that received higher education and patients that have higher health skills more frequently make use of a patient portal [32]. These patients are possibly better equipped to interpret their medical data.

Owing to the nature of our database to capture adverse consequences, we were unable to examine and report on positive experiences of patients concerning real-time disclosure. However, in further research, it would be valuable to examine positive experiences with real-time disclosure to indicate what good it could potentially bring, which aspect benefits patients most and how it benefits them.

Generalizability of study results is limited because of possible selection bias and information bias. In the survey, certain departments are overrepresented; therefore, this study is not representative of the hospital population. The databases were analyzed anonymously, and the majority of issues were brief and did not specify patient characteristics, which made it impossible to differentiate between the experiences of patients with severe or benign illnesses or between patients with acute or chronic illnesses. Issues at the helpdesk were registered by 3 different helpdesk assistants, and the patients’ emotions were not consequently addressed; therefore, this database could not contribute to exploring the emotional consequences of real-time disclosure.

### Conclusions and Recommendations

We showed that the number of severe negative experiences resulting from real-time access to a patient portal was limited in relation to the number of patients that logged onto the portal. We did see some negative experiences with real-time disclosure resulting in patient anxiety, worry, confusion, or panic and incidentally anger, but these accounts did not seem to lead to harmful adverse consequences. The psychological impact originated from the unawareness of disclosure in real time, confrontation with unannounced information, disclosure of adverse results, inability to interpret results, and unavailability of health care professionals for additional explanation soon after disclosure.

These findings justify a policy that minimizes risks of real-time disclosure. Negative consequences that can occur from real-time disclosure of medical information can be mitigated by adequate preparation and instruction of patients concerning the various functionalities of the patient portal, real-time disclosure of test results in particular. To prevent anxiety, worry, panic, and confusion, it is essential that health care professionals are quickly available for questions or that an agreement has been made as to when health care professionals will be available. Moreover, it is of the utmost importance that patients and health care professionals discuss what patients can expect, what the follow-up procedure will look like, and also whether real-time insight into one’s medical record is desirable or whether it is preferable to wait for in-person consultation. The results of this study are helpful in providing insight into the experiences of patients with real-time disclosure and highlight the ways in which negative consequences of real-time disclosure can be mitigated. Further research is needed to identify best practices for discussing real-time disclosure with patients and arranging care systems in a manner suitable for this new way of provision of medical information.

## Appendix

Multimedia Appendix 1 Pop-up shown to patients upon entering the results section.

Multimedia Appendix 2 Number of patients that logged in to the patient portal at least once.

## Abbreviations

e-consults: electronic consults
eHealth: electronic health
EHR: electronic health record
NIP: notifications of incidents in patient care
UMCU: University Medical Center Utrecht
WHO: World Health Organization
