# Supplementary figures and images for: Real-Time Access to Electronic Health Record via a Patient Portal in a Tertiary Hospital: Is it Harmful? A Retrospective Mixed Methods Observational Study
Source: J Med Internet Res. 2020 Feb 11;22(2):e13622. doi: 10.2196/13622 (PMC7055752; doi:10.2196/13622)

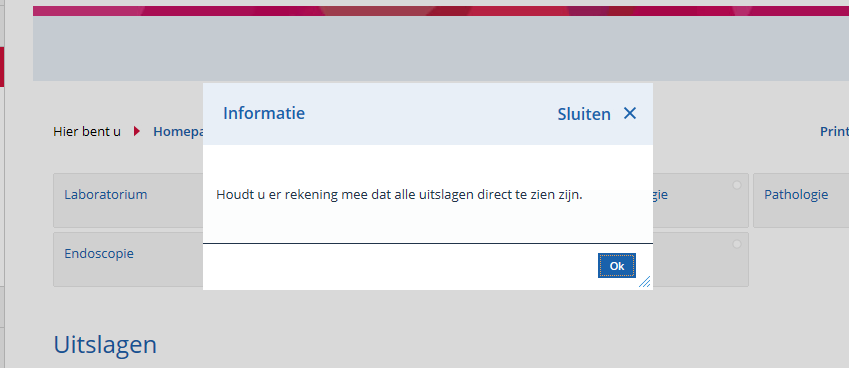

Supplement: Multimedia Appendix 1 [file jmir_v22i2e13622_app1.png]

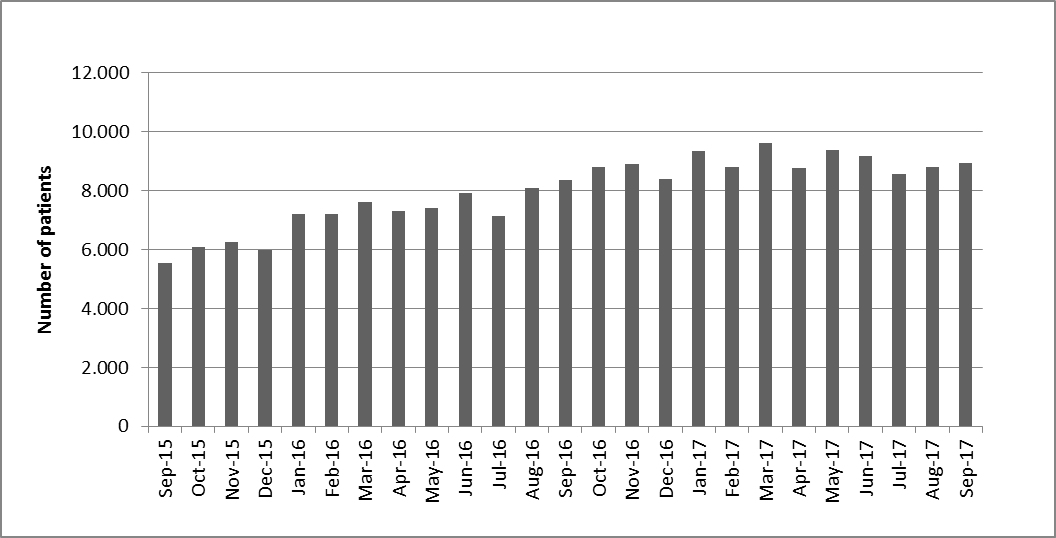

Supplement: Multimedia Appendix 2 [file jmir_v22i2e13622_app2.png]
